# Peer Review of “Interpreting the Estimand Framework From a Causal Inference Perspective”

**DOI:** 10.2196/98126

**Published:** 2026-05-22

**Authors:** Hao Wu

**Affiliations:** 1 Michigan State University, East Lansing, MI, United States

**Keywords:** causal inference, clinical trial, estimand, intercurrent event, treatment effect

*This is a peer review report for “Interpreting the Estimand Framework From a Causal Inference Perspective.”*

## Round 1 Review

This manuscript [1] provides a pedagogical interpretation of the International Council for Harmonisation of Technical Requirements for Pharmaceuticals for Human Use (ICH) E9 framework through the lens of the potential outcomes causal inference framework. The author translates the 5 attributes and intercurrent event strategies into formal statistical notation, primarily using simple randomized trial settings and linear models. The paper is largely conceptual and expository in nature, aiming to improve methodological clarity rather than introduce new causal methodology.

Within the context of *JMIRx Med* as an overlay journal for preprints, the manuscript is generally coherent, technically correct at a basic level, and suitable as an educational or perspective-style contribution, though it would likely fall short of expectations for novelty, depth, or rigor in a specialist statistics or causal inference journal.

### Major Comments

1. The manuscript repeatedly states that it “interprets” the ICH E9 framework, but in practice, it mostly rephrases ICH E9 concepts using potential outcomes notation. Readers would more likely expect to see discussions on limitations, ambiguities, or contested aspects.
2. While pedagogical simplicity may be intentional, several aspects risk being misleading if read uncritically. For example, conditioning on posttreatment variables (section 3.6) is introduced without adequate warning about collider bias or causal ordering issues, and the discussion of principal stratification glosses over identification challenges, relying on brief mentions of Bayesian methods without clarifying assumptions. These are not fatal flaws, but the author should be more explicit about what is heuristic versus formally justified.

## Abbreviations

ICH: International Council for Harmonisation of Technical Requirements for Pharmaceuticals for Human Use
